# Spectrum Fitting Approach for Passive Wireless SAW Sensor Interrogation Using Software-Defined Radio

**DOI:** 10.3390/mi16060656

**Published:** 2025-05-29

**Authors:** Shihao Wang, Qi Wang, Guopeng Zhu, Lei Liu, Xinning Cao, Tingxin Ren, Yue Zhou, Hao Jin

**Affiliations:** 1 Information & Telecommunications Company, State Grid Shandong Electric Power Company, Jinan 250013, China; wangshihao711@163.com (S.W.); wang_a_qi@163.com (Q.W.); zhuguopeng@163.com (G.Z.); xinningcao@126.com (X.C.); 17862707957@163.com (T.R.); 2 State Grid Shandong Electric Power Company, Jinan 250013, China; liulei098332@163.com; 3 College of Information Science & Electronic Engineering, Zhejiang University, Hangzhou 310027, China; 12431009@zju.edu.cn

**Keywords:** SAW sensor interrogator, software defined radio, passive wireless sensing, spectrum fitting approach, SAW sensing

## Abstract

Passive wireless surface acoustic wave (SAW) sensors are widely adopted for monitoring the safety status of industrial equipment due to their compact size and maintenance-free operation. Replacing traditional discrete-component interrogators with software-defined radio (SDR) architectures offers lower cost and greater flexibility. However, conventional frequency estimation methods often rely on iterative algorithms with high computational complexity, limiting their real-time applicability. This paper presents an SAW sensing system based on an SDR platform and a non-iterative spectrum-fitting method for SAW frequency measurement. The feasibility of the proposed method is theoretically analyzed, and its performance under different window functions and length of fast Fourier transform (FFT) configurations is evaluated through simulations and experimental measurements. The results demonstrate a favorable trade-off between time efficiency and SAW frequency measurement accuracy. Compared to traditional approaches, the proposed method reduces complexity while maintaining ± 3kHz peak-to-peak accuracy with only 4096-point FFT length according to experimental results.

## 1. Introduction

Passive wireless SAW sensors have demonstrated versatile applications across discrete sensing domains, including but not limited to temperature monitoring of industrial equipment [1,2,3], strain detection in structural health monitoring systems [1,4], and gas concentration measurement for environmental safety [5,6]. Their advantages such as compact size, maintenance-free operation, and robustness in harsh environments make them particularly suitable for these applications. Compared to delay-line SAW devices, one-port SAW resonators offer higher sensitivity and simplified architecture with reduced interdigital transducer (IDT) size [7]. Their narrow bandwidth and high quality factor (Q-factor) enhance interference rejection and echo signal strength, thereby achieving superior reader sensitivity. This makes them particularly suitable for applications where high-capacity sensor encoding arrays are not required.

Numerous approaches have been developed to extract the SAW resonant frequency from its echo signals, among which FFT remains the predominant choice [8,9,10,11,12,13]. Nevertheless, the FFT spectrum resolution is fundamentally constrained by two factors, the finite Q-factor of the SAW resonator and the limited sampling rate of the interrogation system, resulting in truncated echo signals that preclude high-resolution spectral analysis.

Although existing methods that incorporate FFT with frequency estimation algorithms can achieve high resolution, their reliance on iterative computations imposes significant time overhead [14,15]. To circumvent this limitation, we propose a non-iterative spectrum fitting method that provides practically sufficient resolution for SAW sensing applications. Despite slightly lower precision than iterative-based estimation approaches, its reduced computational complexity ensures applicability for real-time SAW sensing.

The conventional architecture for SAW sensor signal acquisition, implemented through discrete RF components [16] like LNAs, PAs, oscillators, frequency synthesizers, mixers, and analog-to-digital/digital-to-analog (AD/DA) converters, presents inherent limitations in both cost and operational adaptability. In recent years, with the maturation of SDR technology, these discrete components have been integrated into a single chip. For instance, the AD9363 transceiver from Analog Device covers a wide frequency range from 325 MHz to 3.8 GHz and supports tunable channel bandwidths up to 20 MHz, making it highly suitable for demodulating and processing SAW echo signals. SDR significantly simplifies system architecture, reduces hardware complexity and cost, [17,18,19,20,21], and, when combined with ZYNQ-based embedded systems, establishes a reconfigurable platform for optimized SAW signal analysis across heterogeneous application scenarios.

**Figure 1:**
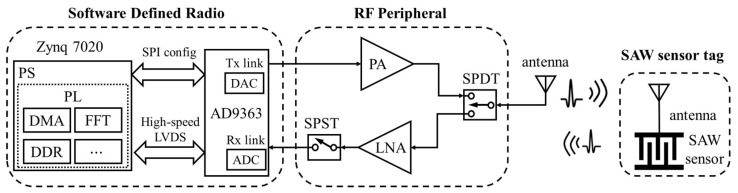
Fundamental SAW interrogator architecture based on SDR.

Figure 1 illustrates the fundamental SAW interrogator architecture based on the SDR system using AD9363. To extend the wireless operating range, external PA and LNA have been incorporated into the base SDR platform. A single-pole double-throw (SPDT) RF switch controlled by FPGA is required to achieve rapid switching between transmit and receive sequences during SAW sensor interrogation. Additionally, a single-pole single-throw (SPST) switch positioned at the LNA output enhances system isolation.

The AD9363 transceiver operates in Frequency Division Duplex (FDD) independent mode. It first generates a burst signal with a programmable center frequency, bandwidth, and duration to excite the SAW sensor tag, and then immediately switches to the receive mode to capture the returning SAW echo signal. The echo signal is downconverted to intermediate frequency (IF), sampled by the 12-bit analog-to-digital converter (ADC) operating up to 60 MSPS, and filtered by the digital finite impulse response (FIR) filter integrated in the AD9363. The sampled data are ultimately transferred via the high-speed LVDS interface to the ZYNQ’s programmable logic (PL), where it is written into the double data rate (DDR) memory through direct memory access (DMA), enabling the processing system (PS) to access it.

## 2. Theory and Simulation of the Proposed Method

### 2.1. Theoretical Analysis

The proposed method first captures *N* periods of SAW echo signals with $L_{sig}$ points in the IF band using the SDR system in Figure 1; the *i*-th (0≤i<N) period of the echo signal is represented as $s_{i}\left[m\right]$, where the index *m* satisfies $0\leq m<L_{\mathrm{sig}}$. The signal is then multiplied by the window function w[m] and zero-padded to a total length of $L_{tot}$, where $L_{tot}=N\times L_{sig}$. Mathematically, the zero-padded signal can be expressed as follows:

(1) $$x_{i}\left[n\right]=\left\{\begin{array}{cc} {\tilde{s}}_{i}\left[n-\left(i-1\right)L_{sig}\right],\; & \left(i-1\right)L_{sig}\leq n<i\times L_{sig}\\ 0, & \mathrm{otherwise} \end{array}\right.$$

where ${\tilde{s}}_{i}\left[m\right]=s_{i}\left[m\right]\times w\left[m\right]$ is the windowed signal of one period, and the index *n* ranges from 0 to $\left(L_{tot}-1\right)$. For each sequence $x_{i}\left[n\right]$, the windowed SAW echo signal ${\tilde{s}}_{i}\left[m\right]$ is assigned starting from $n=i\;\cdot \;L_{\mathrm{sig}}$, while all other positions are zero-padded. Thus, summing over all $x_{i}\left[n\right]$ is equivalent to concatenating the data from *N* acquisitions, resulting in a long-term time domain signal x[n]:

(2) $$x\left[n\right]=x_{0}\left[n\right]+x_{1}\left[n\right]+...+x_{N-1}\left[n\right],\;0\leq n<L_{tot}$$

Let $X_{i}\left[k\right]$ denote the spectrum of $x_{i}\left[n\right]$, where *k* represents the discrete frequency index. The spectrum of the first period signal $x_{0}\left[n\right]$ can be expressed as follows:

(3) $$X_{0}\left[k\right]=\sum_{n=0}^{L_{tot}-1}x_{0}\left[n\right]\;\cdot \;e^{-j\frac{2\pi }{L_{tot}}\;\cdot \;k\;\cdot \;n}=\sum_{n=0}^{L_{sig}-1}{\tilde{s}}_{0}\left[n\right]\;\cdot \;e^{-j\frac{2\pi }{L_{tot}}\;\cdot \;k\;\cdot \;n}$$

For the remaining segments $x_{i}\left[n\right]$, the signal can be considered a $i\times L_{sig}$-point circular right-shifted version of the first period. Therefore, their spectrum $X_{i}\left[k\right]$ can be derived using the FFT shift property by multiplying with a phase rotation factor:

(4) $$X_{i}\left[k\right]=e^{-j\frac{2\pi k}{L_{tot}}\;\cdot \;i}\times \sum_{n=0}^{L_{sig}-1}{\tilde{s}}_{i}\left[n\right]\;\cdot \;e^{-j\frac{2\pi }{L_{tot}}\;\cdot \;k\;\cdot \;n}$$

Let X[k] denote the spectrum of x[n]. It can be obtained through linear superposition of $X_{i}\left[k\right]$. In each acquisition, the measured frequency and phase of the echo signal are inevitably affected by measurement noise and environmental disturbances, leading to random fluctuations around the true frequency in the spectrum. These variations are preserved and superimposed in the concatenated signal spectrum. Generally, such random deviations can be modeled as Gaussian random variables with a mean μ and variance $\sigma^{2}$. The corresponding probability density function (PDF) of the spectral distribution is given by

(5) $$p\left(f\right)=\frac{1}{\sqrt{2\pi \sigma^{2}}}\exp\left(-\frac{{\left(f-\mu \right)}^{2}}{2\sigma^{2}}\right)$$

where *f* denotes the frequency variable of the FFT spectrum. Taking the logarithm of the spectral amplitude yields a downward-opening parabola:

(6) $$\ln p\left(f\right)=-\frac{1}{2}\ln\left(2\pi \sigma^{2}\right)-\frac{{\left(f-\mu \right)}^{2}}{2\sigma^{2}}$$

Therefore, performing a quadratic fit on the logarithmic spectrum, the parabola vertex corresponds to the mean μ, which in turn represents the estimated frequency $\hat{f_{0}}$.

**Figure 2:**
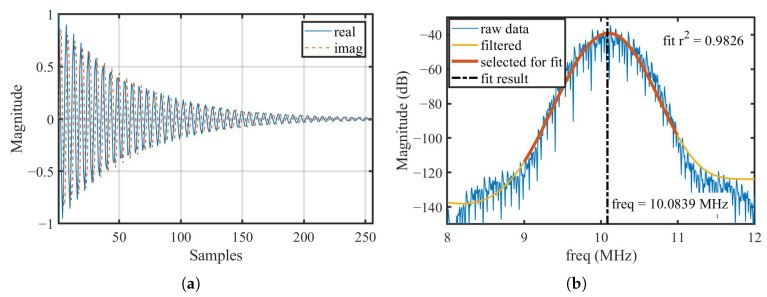
(**a**) A single-cycle 10.100MHz quadrature sinusoidal signal with exponential decay envelope employed in the simulation. (**b**) FFT spectrum of an 8192-point concatenated time-domain signal incorporating random amplitude noise and random frequency deviation: raw data, filtered output, and fitting results.

Figure 2 visualizes the theoretical analysis above, where Figure 2a is a single-period 256-point 10.100 MHz quadrature sinusoidal signal with an exponentially decaying envelope generated in MATLAB R2022b that simulates the SAW echo signal in IF band, and Figure 2b is the FFT spectrum of the 8192-point concatenated signal; each segment of the concatenated signal incorporates random amplitude noise and random frequency deviation. The sample rate ($f_{s}$) in the simulation was set to 60 MSPS to match that of the AD9363. The raw data of the FFT spectrum exhibit high spikes induced by signal concatenation. To enable accurate SAW frequency calculation, a median filter was employed to suppress these spikes, and an infinite impulse response (IIR) low-pass filter (LPF) was employed to further smooth the spectrum.

In Figure 2b, the yellow curve represents the spectrum after filtering, with the red segment indicating the data selected for curve fitting within the range of 9– 11 MHz. A parabolic fitting is applied to this selected region, and the resonant frequency of the SAW device is determined by the x-coordinate of the parabola’s vertex.

For conventional FFT analysis without signal concatenation and spectrum fitting, the frequency measurement deviation is calculated as follows:

(7) $$df=\frac{f_{s}}{L_{sig}}=\;234\;k\mathrm{Hz}$$

Typically, SAW temperature sensors fabricated on YX-cut quartz substrates exhibit a sensitivity of approximately 10 kHz/°C [22]. Such a coarse frequency resolution would render direct measurements impractical. However, by employing the spectral fitting method proposed in this work, the frequency estimation error, as illustrated in Figure 2b, is reduced to only 16.1 kHz relative to the actual signal frequency, closely approaching the intrinsic sensitivity of the SAW temperature sensor.

It was observed that the spectrum fitting results exhibited a certain degree of random jitter in each trial. To further investigate the influence of parameters such as the signal length ($L_{sig}$) and the total concatenated length ($L_{tot}$) on measurement accuracy, and to optimize the precision of the estimation, a detailed simulation study is presented in the following subsection.

### 2.2. Measurement Error Simulation

**Figure 3:**
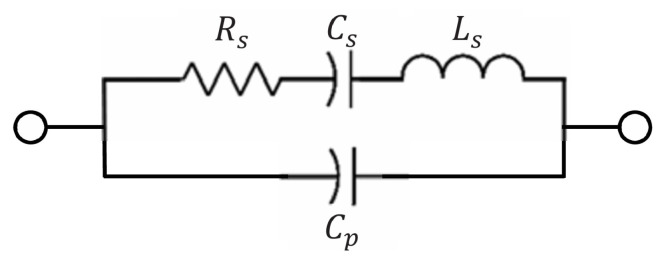
RLC model for single-port SAW resonator.

The duration of the echo signal is determined by the quality factor (*Q*) of the SAW device; a higher *Q* allows a longer portion of the echo to be sampled. The electrical model of a one-port SAW resonator can be represented by the series RLC circuit [10] shown in Figure 3. The time constant τ and the *Q* factor of the circuit are given by

(8) $$\begin{array}{cc} \tau & =\frac{L_{s}}{R_{s}} \end{array}$$

(9) $$\begin{array}{cc} Q & =\frac{1}{R_{s}}\sqrt{\frac{L_{s}}{C_{s}}}=\frac{\tau }{\sqrt{L_{s}C_{s}}}=\tau \omega_{0} \end{array}$$

where $L_{s}$, $C_{s}$, and $R_{s}$ denote the equivalent inductance, capacitance, and resistance, respectively, and $\omega_{0}$ is the natural angular resonant frequency.

Typical SAW resonators exhibit a quality factor (*Q*) of approximately 8000. For an SAW sensor operating at 915 MHz, this corresponds to a time constant of approximately 1.4 μs. It takes roughly 2.3τ for the signal amplitude to decay to 10% of its initial value. Multiplying this duration by the sampling rate yields approximately 193 sampling points.

**Figure 4:**
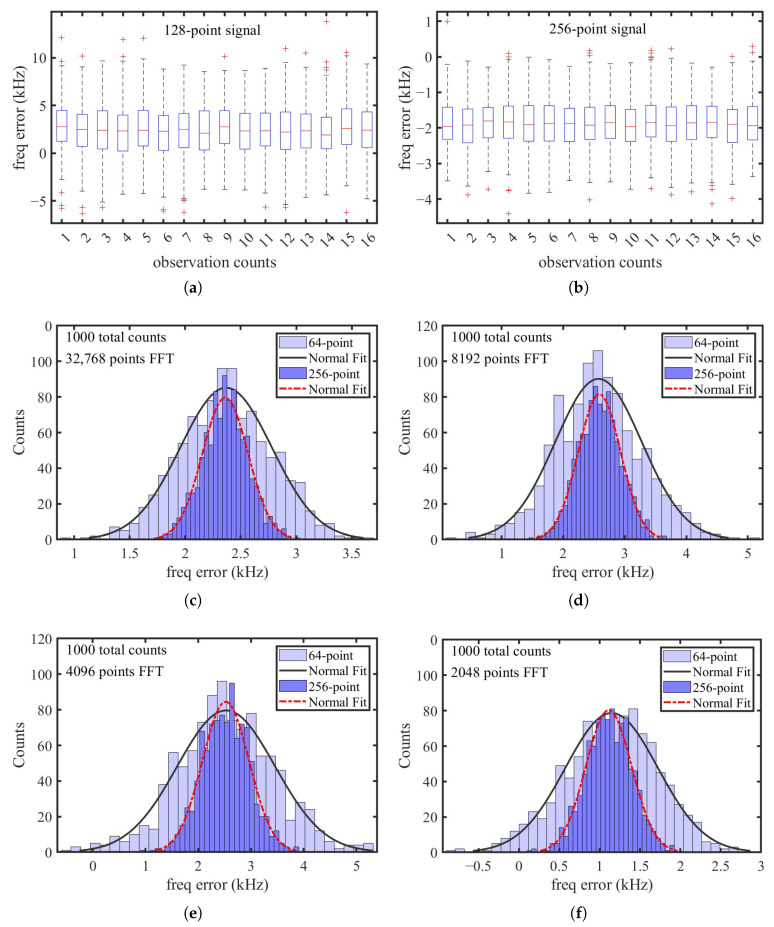
Simulated frequency measurement error using the proposed spectrum fitting method: (**a**) 128-point and (**b**) 256-point SAW echo signal with 16 observation counts; (**c**–**f**) measurement error distributions for FFT lengths of 32,768 points, 8192 points, 4096 points, and 2048 points with 64-point and 256-point median filtering.

In the simulation, exponentially decaying signals with 128 and 256 points were generated to model variations in the resonant frequency and quality factor among different SAW sensors. A total of 16 groups of statistical observations were performed, with each group comprising 256 trials. In each trial, a time-domain signal was constructed by concatenating multiple segments with random frequency, amplitude, and phase noise, followed by spectral fitting. The statistical results are presented as box plots in Figure 4a,b.

As observed from the simulation results, signals with fewer sampling points exhibit significantly larger measurement fluctuations and a higher occurrence of outliers. This necessitates median filtering prior to use, which inevitably compromises the system’s temperature response time. In contrast, when the signal length increases to 256 points, all outliers are confined within 10 kHz, suggesting that the system could theoretically achieve a resolution better than 1 °C without the need for filtering. This makes it particularly suitable for applications involving rapid temperature variations.

For short signals with only 128 points each period, further simulations were conducted to evaluate the impact of different median filter window sizes on measurement accuracy, as well as the effect of varying the total concatenated length (i.e., the FFT size). The simulation results are summarized in Figure 4c–f. This evaluation provides practical guidance for applying the proposed method to measure the resonant frequency of SAW devices with either higher operating frequencies or insufficient quality factors.

In Figure 4, the light-blue distribution represents the frequency measurement error after applying a 64-point median filter, while the dark-blue distribution corresponds to that after a 256-point median filter. The black solid line and red dashed line indicate the normal distribution fittings of the respective measurement errors.

It should be noted that the spectral density varies with different FFT lengths, necessitating adjustments to the median filter window size and the cutoff frequency of the IIR filter used in the FFT spectrum filtering. The detailed filtering parameters are summarized in Table 1. In addition, the mean, standard deviation (Stdev.), and 99% confidence intervals of the frequency measurements, obtained with a 256-point median filter under different FFT lengths, are also listed in the table.

Benefiting from the strategy of concatenating multiple rounds of sampled signals followed by unified spectral fitting, both the effective signal and noise components from each acquisition are superimposed in the frequency domain. If the noise is ideally Gaussian-distributed, as assumed in Equation (6), the true resonant frequency of the SAW resonator can be statistically estimated. This advantage is not available in traditional frequency estimation methods that rely on a single acquisition of the SAW echo signal. The simulation results indicate that, after applying median filtering and appropriately selecting the filter parameters, a short signal with only 128 points per cycle requires merely 2048 sampling points to achieve a peak-to-peak measurement error of approximately 1.5 kHz. This level of accuracy is fully sufficient for traditional one-port resonator-based SAW temperature sensors. Furthermore, a shorter FFT length implies that fewer periods need to be sampled in the time domain. Therefore, from a theoretical perspective, the proposed method can not only maintain sufficient frequency resolution but also effectively reduce computational resource consumption while achieving a faster temperature measurement response.

The computational cost of the proposed method can be evaluated as follows. The method consists of two main stages: data acquisition and spectral fitting. During acquisition, the ARM processor applies a window function to the acquired SAW echo signal, requiring a total of $L_{\mathrm{tot}}$ floating-point operations. However, these operations are effectively hidden by employing a double-buffering strategy, allowing them to be executed concurrently with SAW excitation and echo reception. In the second stage, the FFT is performed in hardware, while the software handles median filtering and curve fitting on the resulting spectrum. Assuming the fitting region contains $L_{\mathrm{fit}}$ points and a median filter of window length *k* is applied, the computational complexity is approximately $L_{\mathrm{fit}}\;\cdot \;k\log k$ using the quicksort algorithm. For the parabolic fitting using the least-squares method, the design matrix X, its transpose $X^{T}$, the Gram matrix $X^{T}X$, and its inverse ${\left(X^{T}X\right)}^{-1}$ can be precomputed for a given fitting range. Therefore, during the fitting process, only the computation of $X^{T}y$ is required, reducing the number of floating-point operations to $3\;\cdot \;L_{\mathrm{fit}}$. This computational cost can be considered negligible compared to the median filtering step.

## 3. Experimental Setup and Results

### 3.1. Implementation of the SDR Interrogator

**Figure 5:**
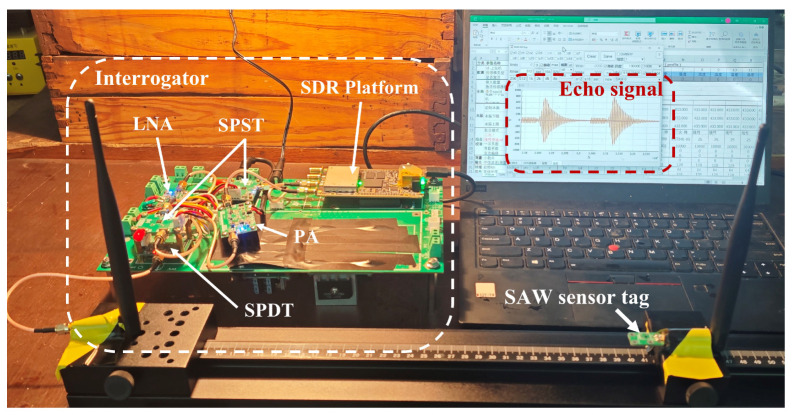
Photograph of interrogator hardware and experimental setup for wireless test.

To experimentally validate the proposed method, an interrogator hardware prototype for wireless test was developed based on the SDR architecture shown in Figure 1. A photograph of the experimental setup is presented in Figure 5. The LNA employs the TQP3M9036 chip, providing approximately 20 dB of gain and an ultra-low noise figure of 0.45 dB at 915 MHz. The PA module delivers an output power of up to 1 W within the same frequency band.

Chirp signals are widely employed in SAW sensor interrogation [21,23,24] due to their advantages in efficiently exciting resonant responses and enhancing signal-to-noise ratios (SNRs). According to [25], when the excitation waveform duration exceeds 15 μs, the maximum amplitude and duration of the SAW echo signal can be achieved. Therefore, a chirp signal comprising 1024 points, corresponding to approximately 17 μs, was selected.

The chirp bandwidth requires balancing trade-offs: If the bandwidth is excessively large, the spectral energy is spread over a wide range, resulting in inefficient coupling with the narrowband SAW resonator and thus degrading the wireless interrogation distance. Conversely, if the bandwidth is too narrow, it becomes necessary to frequently adjust the local oscillator (LO) frequency to track a wide range of sensor responses, which involves re-locking the phase-locked loop (PLL) in the AD9363 and introduces additional time overhead. Moreover, a narrower bandwidth is more difficult to noise disturbances, potentially leading to signal loss. Furthermore, if the local oscillator (LO) frequency is not well aligned with the current resonant frequency of the SAW sensor, a narrower bandwidth makes it more difficult to capture the echo signal. Based on experimental evaluations, a chirp bandwidth of 500 kHz was finally adopted to balance energy efficiency, frequency tracking capability, and system robustness. After determining the length and bandwidth of the chirp signal, it can be generated via a direct digital synthesis (DDS) method in the embedded system.

### 3.2. Wireless Measurement of SAW Echo Signals

**Figure 6:**
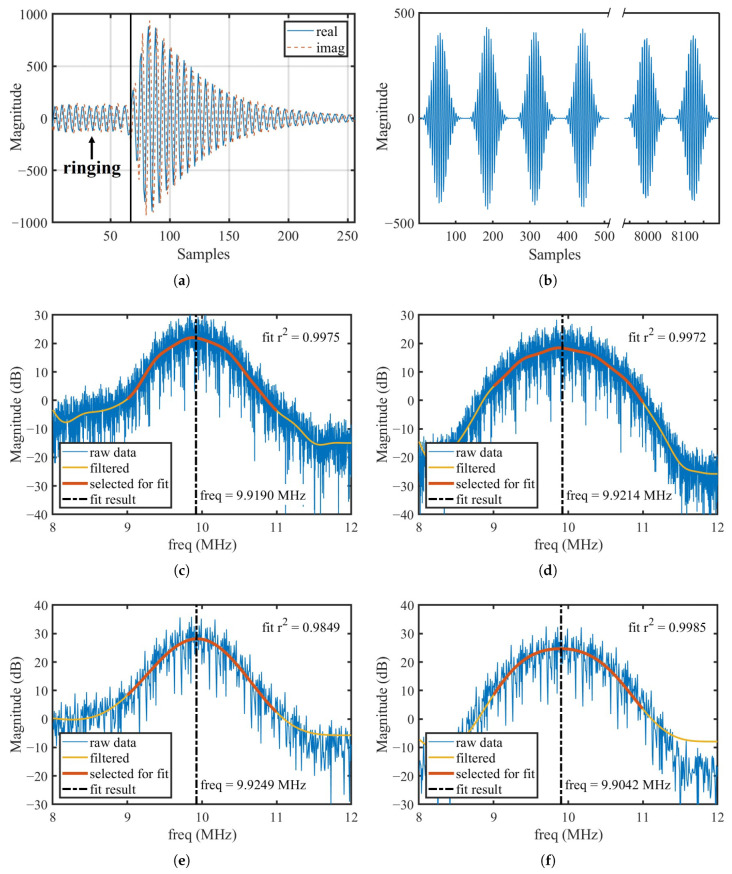
(**a**) Measured SAW echo signal of single peroid and (**b**) 8192-point signal after concatenation; (**c**–**f**) FFT spectrum and fitting results corresponding to 32,768-point Hamming window, 32,768-point Nut window, 8192-point Hamming window, and 8192-point Nut window, respectively.

The developed interrogator was used to perform wireless interrogation of an SAW temperature sensor operating at 911.7 MHz. Two commercial dipole antenna were used for the interrogator and the SAW sensor tag. The captured echo signal and the corresponding spectrum fitting results are shown in Figure 6.

In the short time-domain signal of a single period in Figure 6a, small oscillations occurred during the transmit–receive switching process, primarily due to slight impedance mismatches between the antenna and the PA. After a brief delay of approximately 64 points, the exponentially decaying SAW echo signal appears. To avoid interference, the oscillatory portion is truncated in software. Starting from the 64th point, 128 points are selected as the valid length of the echo signal. After applying a window function, these segments are concatenated as the signal shown in Figure 6b, which is then subjected to FFT analysis and spectrum fitting.

Figure 6c–f compare the spectrum data and fitting results under different window functions and FFT lengths. In each case, the fitting achieves a high coefficient of determination ($r^{2}$), indicating that the parabolic fitting model accurately captures the main spectral characteristics of the SAW echo signal even under practical experimental conditions.

The results demonstrate that the Nut window provides better sidelobe suppression and a broader mainlobe compared to the Hamming window, resulting in a wider parabolic fitting range in the FFT spectrum. As theoretically analyzed in Section 2.1, the concatenation process effectively broadens the spectrum information, enriching the available information and averaging out random noise. Therefore, a window function with a broader mainlobe is more favorable for robust parabolic spectral fitting.

Finally, it is observed that some random fluctuations remain in the fitted frequency results across different window functions and FFT lengths. As in the simulations shown in Figure 4, a median filter can be further applied to the output data to remove outliers and enhance measurement stability.

### 3.3. Frequency Measurement Accuracy

**Figure 7:**
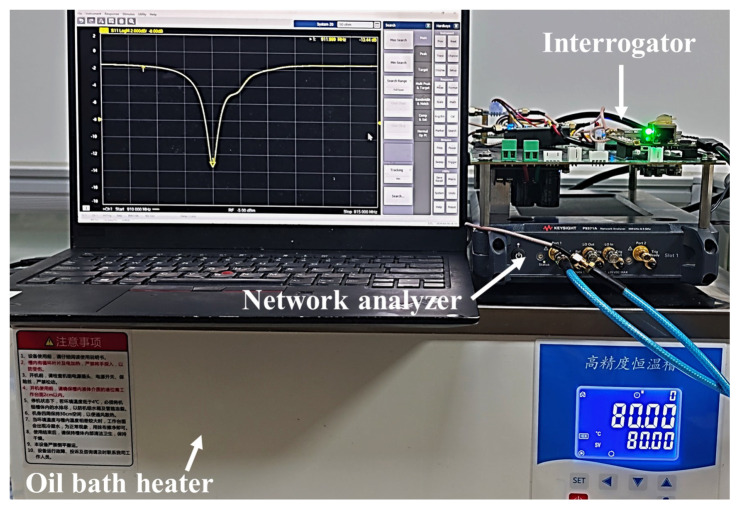
Experimental setup for testing frequency measurement accuracy.

To evaluate the frequency measurement accuracy of the proposed method, an oil-bath heating test environment was constructed, as shown in Figure 7. An SAW temperature sensing unit operating at 915 MHz was tested under both wired and wireless interrogation modes. First, a vector network analyzer (Keysight P9371A, Keysight, Santa Rosa, CA, USA) was used to measure the $S_{11}$ parameter of the SAW sensor at room temperature (R.T.). The frequency corresponding to the minimum of the $S_{11}$ curve was taken as the ground-truth resonant frequency. Subsequently, the same sensor was interrogated under identical conditions using the SDR-based interrogator developed in this study, and the measured frequency was compared with the ground-truth to determine the measurement error. Next, the SAW sensor was immersed in an oil-bath heating environment maintained at 80 °C. After thermal stabilization, the same measurement procedure was repeated to evaluate the system’s performance at elevated temperatures.

**Figure 8:**
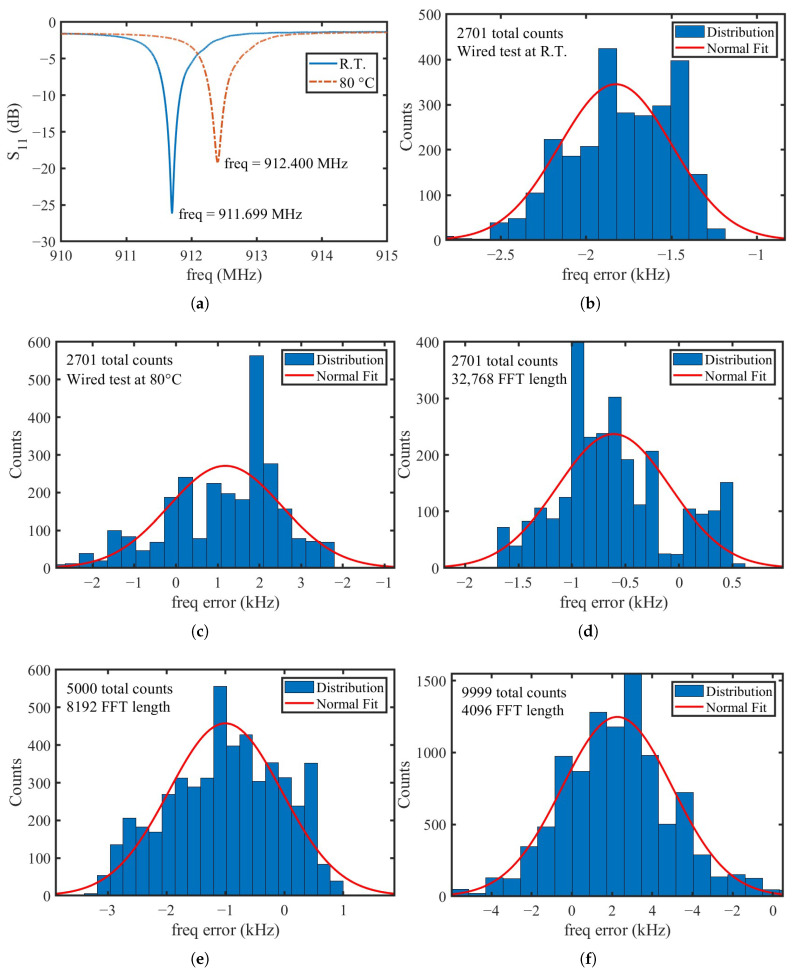
(**a**) $S_{11}$ curves of the SAW resonator measured at R.T. and 80 °C; (**b**) frequency measurement errors under wired interrogation at R.T. and (**c**) 80 °C; (**d**–**f**) frequency measurement errors under wireless interrogation at R.T., corresponding to FFT lengths of 32768, 8192, and 4096 points, respectively.

Figure 8a shows the $S_{11}$ curves and corresponding resonant frequencies of the SAW resonator measured at room temperature and in an 80 °C oil-bath heating environment. As the temperature increased, the resonant frequency shifted upward by approximately 700 kHz, accompanied by a slight degradation in impedance matching.

Due to the shielding effect of the metallic oil-bath chamber, wireless measurements could not be performed inside the narrow environment of the heating chamber. Therefore, a wired interrogation method was used to measure the sensor frequency at both temperatures. The output frequency was smoothed using a 256-point median filter. The distribution of measurement errors is shown in Figure 8b,c. In both measurements, the reader employed an 8192-point FFT and a Nut window that provides a broader fitting bandwidth. A total of 2701 frequency interrogations were conducted. The results show that under controlled isothermal conditions using wired measurement, the peak-to-peak frequency error did not exceed 3 kHz, which is consistent with the simulation results presented above.

Subsequently, the same SAW sensor was wirelessly interrogated at room temperature using the experimental setup shown in Figure 5. The impact of different FFT lengths on the frequency measurement error distribution was investigated, with Figure 8d–f corresponding to FFT lengths of 32,768, 8192, and 4096 points, respectively. The measured peak-to-peak frequency errors under these configurations were approximately 2.5 kHz, 4 kHz, and 6 kHz. Although the measurement error increases slightly as the FFT length decreases, the resulting errors remain within an acceptable range for traditional SAW temperature sensors, considering their typical temperature sensitivity. Moreover, a reduced FFT length implies a faster system response without significantly compromising accuracy, which represents a key advantage of the proposed system.

**Figure 9:**
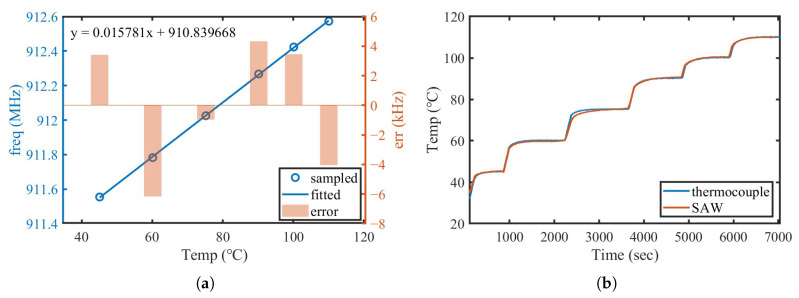
(**a**) Frequency–temperature data from constant-temperature oil-bath calibration and the corresponding fitting error; (**b**) comparison of SAW-based temperature measurement and thermocouple readings.

The proposed interrogator was then calibrated in the oil-bath environment shown in Figure 7. Six temperature points ranging from 45 °C to 110 °C were calibrated. After the oil-bath temperature stabilized, the measured SAW frequency and thermocouple temperature were recorded. The calibration results are shown in Figure 9a, where the measured temperature sensitivity of the SAW sensor was 15.781kHz/°C. The maximum frequency deviation at each point was only −6 kHz, corresponding to approximately 0.38 °C. Based on the obtained calibration parameters, the SAW sensor was then tested in the same environment as the temperature increased from approximately 32 °C to 110 °C. The comparison between the SAW-based measurement and the thermocouple is illustrated in Figure 9b. The SAW temperature response closely matched the thermocouple readings, with minimal noise and deviation, further demonstrating the stability and reliability of the proposed system.

## 4. Discussion and Conclusions

This work presents a software-defined radio (SDR)-based reader system for wireless surface acoustic wave (SAW) sensors, employing a spectrum fitting approach to estimate the resonant frequency with high precision. The proposed system integrates both hardware and algorithmic innovations to address the challenges of low-latency and accurate frequency extraction in narrowband passive SAW devices.

From a theoretical perspective, we derived a mathematical formulation of the spectrum fitting method and demonstrated that the frequency corresponding to the vertex of a parabolic fit in the spectral domain provides a robust and interpretable estimate of the SAW resonant frequency. Through systematic simulations, we analyzed the trade-offs between signal length, filter characteristics, and frequency estimation accuracy. The results indicate that, by applying a properly selected window and filter, a short signal comprising only 2048 samples is sufficient to achieve a peak-to-peak frequency error below 2 kHz.

Experimental measurements further validated the method. First, we evaluated the impact of different window functions on the fitting performance and confirmed that those with broader mainlobes, such as the Nut window, enabled wider and more stable parabolic fitting ranges. Next, we performed wireless measurements at room temperature and wired measurements under controlled oil-bath heating environments at both room temperature and 80 °C. The results showed that the peak-to-peak frequency error remained within approximately ± 1.5 kHz using a 32768-point FFT, and within approximately ± 3 kHz using only 4096 points. These findings demonstrate that the system maintains high accuracy even with reduced FFT lengths, enabling faster response times without significant loss of measurement precision. The system is then calibrated and the SAW sensor demonstrates excellent agreement with thermocouple measurement results, accurately capturing temperature variations with low error.

In conclusion, the spectrum-fitting-based SAW reader system demonstrated herein provides a promising solution for high-resolution frequency tracking using lightweight SDR hardware. It offers a compelling balance between spectral resolution, computational efficiency, and robustness, making it well suited for temperature sensing applications where measurement speed and precision are both critical. Future work will explore multi-sensor addressing schemes and robustness under non-ideal wireless environments.

## Figures and Tables

**Table 1:** Simulated frequency measurement error and filter parameters with different FFT length.

FFT Len	Mean (μ)	Stdev. (σ)	99% Confidence Bound ^1^		Span ^2^	$k_{\mathrm{midF}}$ ^3^	$f_{c}$ ^4^
	**(kHz)**	**(kHz)**	**Lower**	**Upper**	**(kHz)**		
32,768	2.360	0.206	1.831	2.890	1.059	61	0.01
8192	2.589	0.343	1.706	3.472	1.766	31	0.05
4096	2.522	0.444	1.379	3.665	2.287	21	0.07
2048	1.113	0.283	0.383	1.843	1.460	21	0.10

^1^ The units are in kHz. ^2^ Span=(Upperbound−Lowerbound) as measurement error range. ^3^ Median filter window size for FFT spectrum. ^4^ Normalized iir low-pass filter cutoff frequency for FFT spectrum.

## Data Availability

The data supporting the findings of this study are available from the corresponding author upon reasonable request.
